# A Geant4-DNA Evaluation of Radiation-Induced DNA Damage on a Human Fibroblast

**DOI:** 10.3390/cancers13194940

**Published:** 2021-09-30

**Authors:** Wook-Geun Shin, Dousatsu Sakata, Nathanael Lampe, Oleg Belov, Ngoc Hoang Tran, Ivan Petrovic, Aleksandra Ristic-Fira, Milos Dordevic, Mario A. Bernal, Marie-Claude Bordage, Ziad Francis, Ioanna Kyriakou, Yann Perrot, Takashi Sasaki, Carmen Villagrasa, Susanna Guatelli, Vincent Breton, Dimitris Emfietzoglou, Sebastien Incerti

**Affiliations:** 1 Bordeaux University, CNRS/IN2P3, CENBG, UMR 5797, 33170 Gradignan, France; tran@cenbg.in2p3.fr (N.H.T.); incerti@cenbg.in2p3.fr (S.I.); 2 Department of Radiation Oncology, Seoul National University Hospital, Seoul 03080, Korea; 3 Biomedical Research Institute, Seoul National University Hospital, Seoul 03080, Korea; 4 Department of Accelerator and Medical Physics, Institute for Quantum Medical Science, QST, Chiba 263-8555, Japan; sakata.dousatsu@qst.go.jp; 5 Unaffiliated, Melbourne, Australia; nathanael.lampe@gmail.com; 6 Veksler and Baldin Laboratory of High Energy Physics, Joint Institute for Nuclear Research, 141980 Dubna, Russia; dem@jinr.ru; 7 Institute of System Analysis and Management, Dubna State University, 141982 Dubna, Russia; 8 Vinča Institute of Nuclear Sciences, University of Belgrade, 11000 Belgrade, Serbia; ipetrov@vin.bg.ac.rs (I.P.); aristic@vin.bg.ac.rs (A.R.-F.); mdjordjevic@vin.bg.ac.rs (M.D.); 9 Insitituto de Fisica Gleb Wataghin, Universidade Estadual de Campinas, Campinas 13083-859, SP, Brazil; mabernal@unicamp.br; 10 Universite Toulouse III-Paul Sabatier, UMR 1037, CRCT, 31034 Toulouse, France; marie-claude.bordage@inserm.fr; 11 INSERM, Universite Paul Sabatier, UMR 1037, CRCT, 31034 Toulouse, France; 12 Saint Joseph University of Beirut, Research Unit Mathematics and Modeling, Beirut 1004 2020, Lebanon; ziad.francis@usj.edu.lb; 13 Medical Physics Laboratory, Department of Medicine, University of Ioannina, 45110 Ioannina, Greece; ikyriak@uoi.gr (I.K.); demfietz@uoi.gr (D.E.); 14 Institut de Radioprotection et de Sûreté Nucléaire (IRSN), BP17, 92262 Fontenay aux Roses, France; yann.perrot@irsn.fr (Y.P.); carmen.villagrasa@irsn.fr (C.V.); 15 KEK, 1-1, Oho, Tsukuba, Ibaraki 305-0801, Japan; takashi.sasaki@kek.jp; 16 Centre for Medical Radiation Physics, University of Wollongong, Wollongong, NSW 2522, Australia; susanna@uow.edu.au; 17 Clermont Auvergne University, CNRS/IN2P3, LPC, 63000 Clermont-Ferrand, France; breton@clermont.in2p3.fr

**Keywords:** Monte Carlo track structure simulation, Geant4-DNA, DNA damage

## Abstract

**Simple Summary:**

DNA damage caused by ionizing radiation in a human fibroblast cell evaluated by the Geant4-DNA Monte Carlo toolkit is presented. A validation study using a computational geometric human DNA model was then carried out, and the calculated DNA damage as a function of particle type and energy is presented. The results of this work showed a significant improvement on past work and were consistent with recent radiobiological experimental data, such as damage yields. This work and the developed methodology could impact a broad number of research fields in which the understanding of radiation effects is crucial, such as cancer radiotherapy, space science, and medical physics.

**Abstract:**

Accurately modeling the radiobiological mechanisms responsible for the induction of DNA damage remains a major scientific challenge, particularly for understanding the effects of low doses of ionizing radiation on living beings, such as the induction of carcinogenesis. A computational approach based on the Monte Carlo technique to simulate track structures in a biological medium is currently the most reliable method for calculating the early effects induced by ionizing radiation on DNA, the primary cellular target of such effects. The Geant4-DNA Monte Carlo toolkit can simulate not only the physical, but also the physico-chemical and chemical stages of water radiolysis. These stages can be combined with simplified geometric models of biological targets, such as DNA, to assess direct and indirect early DNA damage. In this study, DNA damage induced in a human fibroblast cell was evaluated using Geant4-DNA as a function of incident particle type (gammas, protons, and alphas) and energy. The resulting double-strand break yields as a function of linear energy transfer closely reproduced recent experimental data. Other quantities, such as fragment length distribution, scavengeable damage fraction, and time evolution of damage within an analytical repair model also supported the plausibility of predicting DNA damage using Geant4-DNA.The complete simulation chain application “molecularDNA”, an example for users of Geant4-DNA, will soon be distributed through Geant4.

## 1. Introduction

It is possible to epidemiologically predict the biological effects induced by ionizing radiation in humans by following up studies on atomic bomb survivors or cancer patients treated with radiotherapy. However, a mechanistic evaluation of the subsequent radiobiological effects is necessary to better understand radiation carcinogenesis.

DNA is considered as the most critical target for ionizing radiation, and a challenge still lies today in bridging the gap between the irradiation of cells and how mutations or cell death follow as a consequence of DNA damage and repair [1]. Indeed, a mechanistic understanding of radiation-induced DNA strand breaks and clustered/complex DNA lesions, as well as the great variation and complexity of pathways involved in response to DNA damage, are not fully understood, as recently explained by Keta et al. [2]. Nevertheless, while keeping such limitations in mind, Monte Carlo track structure (MCTS) simulation is considered today as a reliable mechanistic approach for radiobiological studies at the cell scale [3].

Several MCTS codes have been developed so far, such as PARTRAC [4], KURBUC [1], Geant4-DNA [5,6,7,8], TOPAS-nBio [9], and RITCARD [10]. These codes propose independent geometric DNA and damage-repair models based on theoretical approaches or experimental data from the literature.

In the case of Geant4-DNA, which was the first toolkit made available for open access to the community [8], DNA geometry seeded from fractal packing has recently been proposed [11,12,13]. This model was validated by comparing it with predictions of other MCTS codes and experimental data [14,15]. In these previous Geant4-DNA studies, two primary limitations appeared: high computing time required for the simulation, and difficulties tuning simulation parameters that differ between codes. First, the step-by-step (SBS) method, which is typically used in combination with MCTS codes for the simulation of water radiolysis, is extremely slow compared to the physics component of the simulation, requiring several days to model proton irradiation in Geant4-DNA [16]. Moreover, most MCTS codes have tuned their simulation parameters to reproduce experimental data, instead of using the values or settings available in the original papers describing the various models (physics, physico-chemistry, and chemistry) on which they are based.

In this study, we proposed the application of recent developments in Geant4-DNA, including a more accurate electron elastic model [17], calibrated pre-chemical [18] and chemical parameters [19], and the independent reaction time (IRT) approach [20], into the “molecularDNA” Geant4-DNA example, which was initially developed by Lampe [11]. In order to validate this work, DNA damage induced in a simplified human fibroblast cell was evaluated using the proposed changes and compared with experimental data.

## 2. Materials and Methods

### 2.1. DNA Geometry

To model the complex double helix structure of DNA geometry in Geant4-DNA, Lampe et al. [11,12,13] proposed a simplified geometric DNA model based on a fractal structure. Here, a Python script was used to generate the fractal geometry, which allowed for some flexibility in the overall structure based on the initial curve the fractal was seeded with. The “molecularDNA” application itself is user-friendly and allows the geometries and test-damage parameters to be set using macro commands.

**Figure 1:**
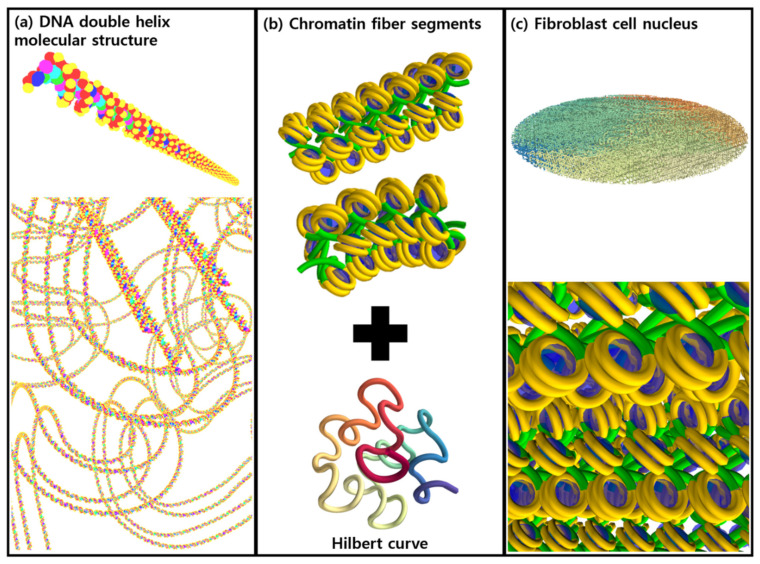
(**a**) Schematic illustration of the molecular structure of the DNA double helix. The spheres represent adenine (C_5_H_5_N_5_, blue), thymine (C_5_H_6_N_2_O_2_, magenta), guanine (C_5_H_5_N_5_O, green), cytosine (C_4_H_5_N_3_O, cyan), sugar (C_5_H_10_O_4_, deoxyribose, red), and phosphate (H_3_PO_4_, yellow), respectively. (**b**) Simplified chromatin fiber segments (straight and turned) and a unit of Hilbert curve. (**c**) The modeled fibroblast cell nucleus.

The DNA geometry is provided in two parts: first, the geometrical configuration of the nucleotide bases and the sugar-phosphate backbone in a curved or straight chromatin segment; and second, the overarching macrostructure of the DNA (here, a fractal Hilbert curve [21]), as shown in Figure 1. One way to think of this is that one file is used to describe a straight or turned DNA segment, and a second describes how they join together to fit into a cell.

First, a chromatin segment was defined in an input file containing the shape, position, and size of the DNA molecules. In order to construct realistic inter-linked chromatin segments, three segment models (“straight”, “turned”, and “turned-twisted” geometries) were produced. The fractal structure of the chromosome was then generated from the Hilbert curve, which is typically used for continuous fractal space-filling [21]. The iteration of the Hilbert curve resulted in a more complex but continuous chromatin fiber. To shape the cell nucleus, a spherical or ellipsoidal mask was used, depending on the cell geometry.

### 2.2. DNA Damage and Scoring

We followed the classification of DNA damage proposed by Nikjoo et al. [22]. Depending on the source of damage, direct damage is induced by physical interactions, and indirect damage occurs from the chemical reaction between a radical and a DNA molecule. Regarding the complexity of the damage, DNA strand breaks are classified as not only single-strand breaks (SSBs) and double-strand breaks (DSBs), but also as complex damage events [22]. In this work, we present a brief description of all the parameters used and the methodology for scoring direct and indirect damages.

First, we defined the types of energy depositions that induce direct DNA damage. PARTRAC proposed a linear damage model in which the likelihood of damage increased from a lower energy threshold, *E*_lower_, of 5 eV to an upper threshold, *E*_higher_, of 37.5 eV [23], based on experiments and parameter adjustments [24,25]. To determine whether an energy deposit impacts DNA, an effective target volume and radius must be defined. Geant4-DNA assumed an effective radius, *R*_direct_, larger than the van der Waals radius of sugar and phosphate (2.28 and 2.63 Å, respectively), in order to consider the energy depositions in the hydration shells of these molecules. The *R*_direct_ value was optimized in a previous Geant4-DNA study [15].

The calculation of indirect damage requires simulation of water radiolysis. In brief, the molecular species generated during the pre-chemical stage undergo not only chemical reactions with the sugar-phosphate backbone, inducing so-called indirect strand breaks, but are also scavenged by each other or by histone proteins. To induce indirect strand breaks, the chemical reaction occurs between the ^•^OH radical and the sugar-phosphate group. In this work, the probability, *P*_OH_, of this reaction to induce an SSB was set to 40.5%, causing approximately 13% of all reactions between DNA and ^•^OH to induce a DSB, which was consistent with previous experimental studies and simulations [13].

To reduce the computational time for radiolysis simulation, two key chemical parameters were identified in a previous study by Sakata et al. [15], as derived from the work of Lampe et al. [13]. It was assumed that all molecular species generated further away than a “radical kill distance”, *d*_kill_, from the DNA molecule would be scavenged by the medium based on their scavenging rate and diffusion rates of radicals [26,27]. This parameter is linked to the end time, *T*_chem_, of the chemical stage by the time taken for an ^•^OH radical to *d*_kill_.

A maximum time step, *dt*_max_, was also used in this study. This idea was initially proposed in previous Geant4-DNA works for the simulation of DNA damage induced by ionizing radiation in E. coli [11,13,28]. The entire chemistry simulation through *T*_chem_ was split into several time steps of maximum duration *dt*_max_, and the reaction times are sampled at each time step [29].

All damage parameters optimized in our previous study [15] are listed in Table 1.

The chemical reaction rates between the DNA molecules and radicals were those proposed by Buxton et al. [30]. In this work, the reactions were added to the Geant4-DNA chemistry constructor “G4EmDNAChemistry_option3” [18,20].

Breaks, regardless of source (direct or indirect), were classified by their complexity. A DSB represented two breaks on opposite strands that were simultaneously induced within the distance *d*_DSB_, taken as 10 base pairs (bp). If the separation distance between two breaks was larger than *d*_DSB_, the breaks were considered as two independent SSBs. We also considered a fragment gap, *d*_s_, that determined the complex SSBs and DSBs. In Geant4-DNA, a *d*_s_ value of 100 bp was used [13]. The complex SSBs were denoted as SSB+ or 2SSB if the damage was located on the same or opposite strands, respectively. The complex DSBs, DSB+, and DSB++ were more important than complex SSBs because they determined irreparable DNA damage in our calculations. DSB+ classification required one DSB and one more break within the *d*_DSB_. Two DSBs within the fragment gap *d*_s_ were classified as DSB++.

In addition, SSBs and DSBs could also be classified as direct damage, denoted as SSB_d_ and DSB_d_; indirect damage, denoted as SSB_i_ and DSB_i_; or mixed damage (one direct and one indirect), denoted as SSB_m_ and DSB_m_, in order to evaluate the protectable DNA damage using the radical scavenger. DSB_hyb_ is a mixed damage classification; however, it was only considered when indirect damage was the core damage of the DSB.

### 2.3. Simulation Configuration for Validation Study Using a Simplified Human Fibroblast Cell

The improvements proposed in our previous works were implemented in the simulations presented here. The physics constructor G4EmDNAPhysics_option2 with the new ELSEPA electron elastic scattering model [17] was used. In the case of the chemistry constructor, the most recent version, G4EmDNAChemistry_option3 including DNA reactions, was selected. This constructor consisted of the Meesungnoen electron thermalization model [31] for thermalized and auto-ionization electrons [19], pre-chemical parameters [18], our IRT method and the corresponding chemical reaction table [20], and other improvements such as the initial displacement of ^•^OH radicals and the electron attachment model [32].

The IRT method approximated the reaction time based on the reaction rate and separation distance between a pair of molecular species independently. This approximation assessed the diffusion of molecular species to calculate the reaction time, but not to track their trajectories. Thus, this approach presented the advantage of much faster simulation times compared to the SBS method. However, the simulation of chemistry kinetics without the presence of all the reactive molecules and diffusion simulations may cause imprecision in simulated DNA damage. This technique was recently implemented in Geant4-DNA and validated for G-values [20].

An octree-node approach was employed to search for neighboring radicals [33]. An octree data structure divides a node into eight smaller nodes. The octrees of this application contained the positions of all the radicals, allowing Geant4-DNA to rapidly identify neighboring reactants around any given DNA molecule.

According to several studies on the geometry of fibroblast cells, such cells are about 2260 μm^3^ in volume [34] and consist of ~6 × 10^9^ base pairs placed in ~500 μm^3^ nucleus [35]. Based on the literature, we considered a simplified geometry of a human fibroblast cell [14], consisting of an ellipsoidal cell nucleus described by the equation $\frac{x^{2}}{{\left(14.2\;\mu m\right)}^{2}}+\frac{y^{2}}{{\left(14.2\;\mu m\right)}^{2}}+\frac{z^{2}}{{\left(5\;\mu m\right)}^{2}}=1$, and surrounded by an ellipsoidal water phantom described by the equation $\frac{x^{2}}{{\left(28\;\mu m\right)}^{2}}+\frac{y^{2}}{{\left(28\;\mu m\right)}^{2}}+\frac{z^{2}}{{\left(5\;\mu m\right)}^{2}}=1$ representing the cytoplasm.

The chromosomes were uniformly distributed in the nucleus based on the Hilbert curve, and the total number of base pairs included in this nucleus was 6.4 Gbp (base pair density of 0.012 bp/nm^3^), which was consistent with the reported base pair density of a mammalian cell (approximately 0.015 bp/nm^3^) [36,37]. Outside the cytoplasm, a near-vacuum was modelled using the Geant4 pre-defined material G4_Galactic.

As a source, ^137^Cs and ^60^Co photon beams, proton beams of 0.3, 0.4, 0.7, 1.0, 1.67, 2.34, 4.0, 7.0, and 50 MeV, and alpha beams with initial energies of 5, 10, and 15 MeV were generated from a plane parallel to the cell nucleus [15].

**Figure 2:**
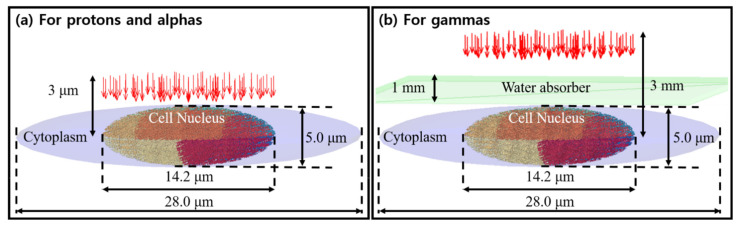
A schematic illustration of the geometrical configuration of the human cell nucleus and source term. Figures adapted from [15].

Rather than reconstruct the exact experimental conditions of each comparison paper down to the petri dish, we compared our results to the LET values reported at the cell entrance. Similarly, we did not account for any substrate. For proton and alpha simulations, the source plane was located 3 μm from the cell center in order to compare with experimental data, as shown in Figure 2a. For gamma irradiation experiments, the cells were suspended in a flask [38], so we included a water absorber of 1 mm thickness, as proposed in the original papers. The source plane was located 3 mm from the cell nucleus, as shown in Figure 2b.

We computed the numbers of total strand breaks, SSBs, and DSBs as a function of LET (values based on the ICRU-90 report [39]), and the corresponding SSB/DSB ratios using the damage parameters for damage scoring given in Table 1.

It should be noted that the measurements of DNA damage shown in this study [40,41,42,43,44,45] used gel electrophoresis techniques such as agarose gel electrophoresis (AGE) [46], constant-field gel electrophoresis (CFGE), and pulsed-field gel electrophoresis (PFGE) [47].

However, this approach presents difficulties in counting fragments of DNA in close proximity to each other; for example, closer than 23 kbp [45], owing to limitations in the detection method. Thus, the yields of distant DSBs, meaning separated by at least 10 kbp between DSBs, were also calculated in this study.

On the other hand, an approach for measuring DNA damage has been proposed via counting of the number of immuno-fluorescent foci such as γ-H2AX [48,49]. This approach also has limitations, such as the non-linear correlation between foci number and DSB yields; however, it was assumed that the ratio of the number of foci to the number of DSB is 1:1 [50]. In addition, the sensitivity was significantly improved compared to that for PFGE [51]. Petkovic et al. [52], Ristic-Fira et al. [53], and Keta et al. [2] evaluated DNA damage by measuring γ-H2AX for gamma ray and proton beams. The details of the experimental data presented in this study are given in Table 2, while the biochemical background is provided elsewhere [54,55].

The calculated DSB results were compared with the experimental data listed in Table 2, as well as the data from Geant4-DNA [14,15,16], PARTRAC [23], and KURBUC [56]. The histogram of the fragment length distribution, which represented the distance between two DSBs, was calculated with 100 Gy of 1 MeV protons, and also was compared with the experimental data of Belli et al. [43] and Campa et al. [45]. Another experimentally accessible quantity was the protectable damage fraction (or scavengeable fraction). This was the fraction of the damage present at infinite dimethyl sulfoxide (DMSO radical scavenger) concentration. Such chemicals can scavenge free molecular species, especially ^•^OH radicals. In the simulation, the protectable damage fraction was the ratio between the DSBs induced by indirect damage and all DSBs. We calculated these fractions as a function of LET and compared them with the experimental data of Ito et al. [57] and Hirayama et al. [58].

### 2.4. Cell Repair Model

Our simulations could be used to predict “early” DNA damage up to 5 ns. However, modeling radiobiological phenomena during the long-term biological stage requires complex mathematical models [59]. Briefly, it has been reported that DSBs induce deletions, translocations, and fusions of DNA when they are not correctly repaired [60]. We did not review the existing repair models and their implementations because they are beyond the scope of this work, and descriptions are available in [61].

However, in the context of the Geant4-DNA collaboration, a repair model based on the advanced mathematical model of Belov et al. [62] was proposed. This model assesses the principal “repair pathways”, known as non-homologous end-joining (NHEJ), homologous recombination (HR), single-strand annealing (SSA), and alternative end-joining mechanism (Alt-NHEJ). The details of these pathways are described in papers by Heyer et al. [63] and Decottignies [64].

In this model, the total yield of DSBs, *N*_0_, as a function of time can be calculated based on the repair pathways as follows:

(1) $$\frac{dN_{0}}{dt}=\alpha \left(L\right)\frac{dD}{dt}N_{ir}-V_{NHEJ}-V_{HR}-V_{SSA}-V_{micro-SSA}-V_{Alt-NHEJ}$$

where *D* is the absorbed dose (Gy), and *α*(*L*) is the DSB induction per dose (Gy^−^^1^ per cell) depending on LET *L*. *N*_*ir*_ is the yield of irreparable DSBs, representing (with our damage scheme) *N*_DSB_^+^ + 2 × *N*_DSB_^++^. *V*_*NHEJ*_, *V*_*HR*_, *V*_*SSA*_, *V*_*micro-SSA*_, and *V*_*Alt-NHEJ*_ are the repair potentials for the NHEJ, HR, SSA, micro-SSA, and Alt-NHEJ repair pathways, respectively. The repair potentials were given via 29 differential equations and 54 parameters, including rate constants for human fibroblasts, in a paper from Belov et al. [62]. This model enabled the calculation of five foci yields, which referred to the response of specific proteins to DSBs [49], such as Ku protein, DNA-PKcs, RPA, Rad51, and γ-H2AX.

In this study, this simplified repair model was employed, which was recently implemented in the “molecularDNA” example by Sakata et al. [15]. The foci yield of γ-H2AX as a function of time was calculated and compared with the experimental data performed by Asaithamby et al. [38].

## 3. Results

As described previously, the computational power for simulating all DNA structures in a human fibroblast cell nucleus and storing damage is still challenging. The “molecularDNA” example with the IRT approach proposed in this study could significantly reduce the calculation time, as shown in Table 3. However, memory consumption remained a burden, particularly for storing DNA damage as a function of LET (e.g., up to 160 GBs for only 10 alpha particles of 5 MeV). Thus, we decided to split the number of particles and run them. The memory was cleaned up for every split, and after all the simulations were completed, the output files were merged and analyzed.

**Figure 3:**
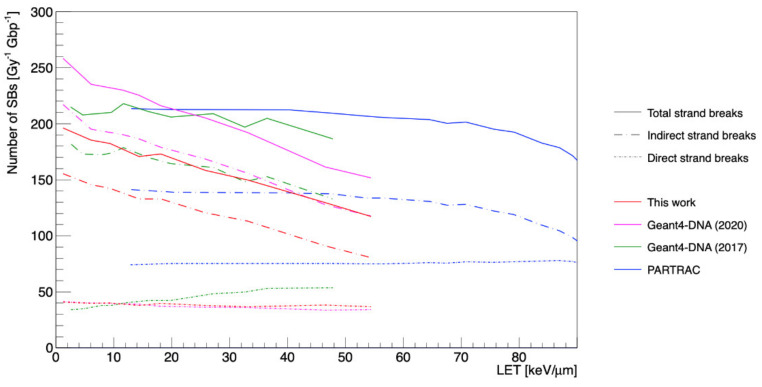
The number of total strand breaks as a function of LET calculated by Geant4-DNA (this work, [15,16]) and PARTRAC [23].

Figure 3 shows the number of total, indirect, and direct strand breaks for incident protons as a function of ICRP-90 LET and in other studies. The number of indirect damages (red dash-dot curve) was remarkably decreased compared to the results of previous studies (Geant4-DNA 2020 [15], Geant4-DNA 2017 [16], PARTRAC [23]), unlike the number of direct damages.

**Figure 4:**
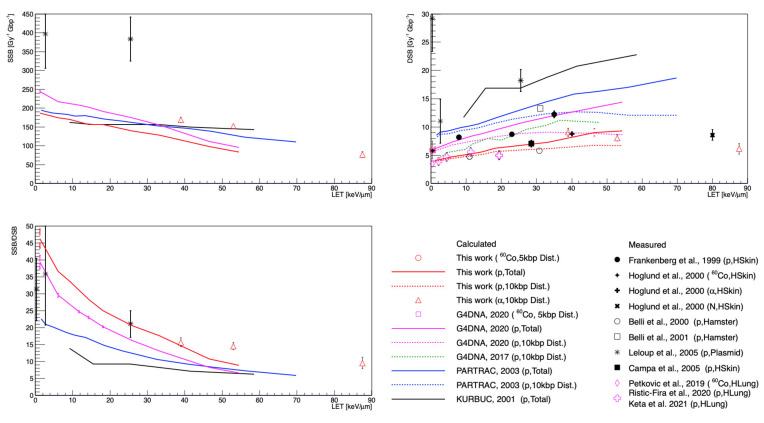
The SSB (**left** upper) and DSB yields (**right** upper), and SSB/DSB ratio (**left** below) as a function of LET for the MCTS simulations and measurements.

Figure 4 shows the calculated SSB and DSB yields and the SSB/DSB ratio for incident gamma rays, protons, and alphas as a function of LET. The DSB yields in this work were lower than those in data from Frankenberg et al. [40] by as much as 62%; however, other experimental data from Hoglund et al. [41] and Campa et al. [45] were consistent with the results of this work, within a 10% difference. In particular, our simulations closely approached the recent experimental data obtained by Petkovic et al. [52] and Keta et al. [2] (magenta diamond and crosses), even though those data were for human lung cells and assumed that the number of foci/cell was equal to the number of DSB/nuclei.

The DSB yields for alpha particles (red triangles) also showed a strong consistency with the already-reported data from Hoglund et al. [41] (black cross). Unlike protons, the DSB yields for alpha particles slightly decreased as a function of LET, due to more complex or clustered DSBs [66], since they overlapped and could not be properly counted one by one, as shown in the experimental data [2].

In addition, higher SSB/DSB ratios were calculated in this study compared with the other simulations. This result closely mirrored the plasmid data of Leloup et al. [44], especially at an LET of 25.5 keV/μm, even though plasmids were not cells.

**Figure 5:**
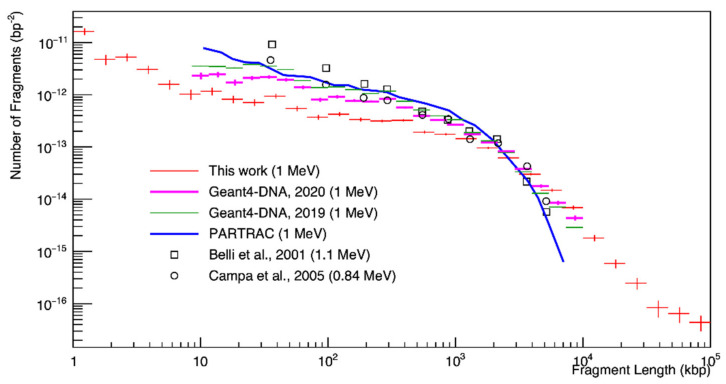
Histogram of the fragment length distribution after 100 Gy irradiation with 1 MeV protons. Simulations (lines) and measurements (symbols) are shown.

The fragment distribution is shown in Figure 5 for 1 MeV proton irradiation. Small fragments were the most frequent, and their distribution decreased as a function of fragment length. The results of this study obtained for 100 Gy underestimated the number of DSBs compared to the PARTRAC simulations and experimental data of Belli et al. [43] (3 MeV incident proton beam and a corresponding energy at beam exit of 1.1 MeV) in Chinese hamster cells (V79), and Campa et al. [45] in human fibroblasts.

**Figure 6:**
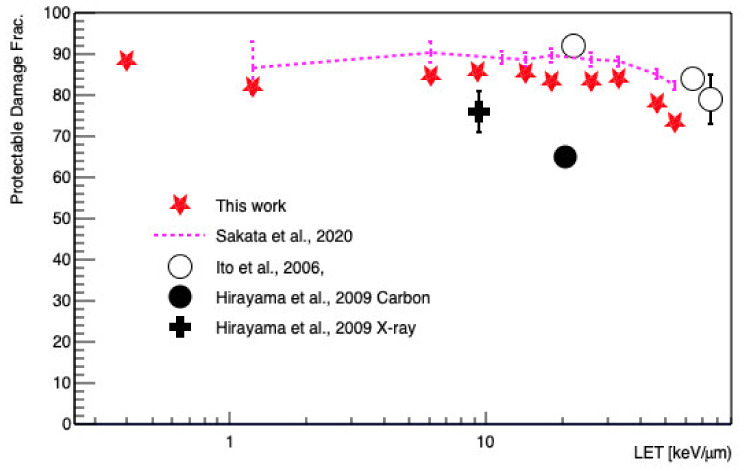
Protectable damage fraction, which is the ratio of protectable DSBs over the total number of DSBs, as a function of LET. Geant4-DNA simulations and measurements are shown.

The protectable (or scavengeable) damage fraction as a function of LET was also calculated, as shown in Figure 6. As reported thus far, the indirect damage fraction was dominant for low LET irradiation and decreases as a function of increasing LET [58]. In an LET range of 1.24–54.41 keV/μm, the results of this work were lower than in a previous study using Geant4-DNA by as much as 10% [15], because this work assessed less indirect damage due to the changes in pre-chemical and chemical stages.

**Figure 7:**
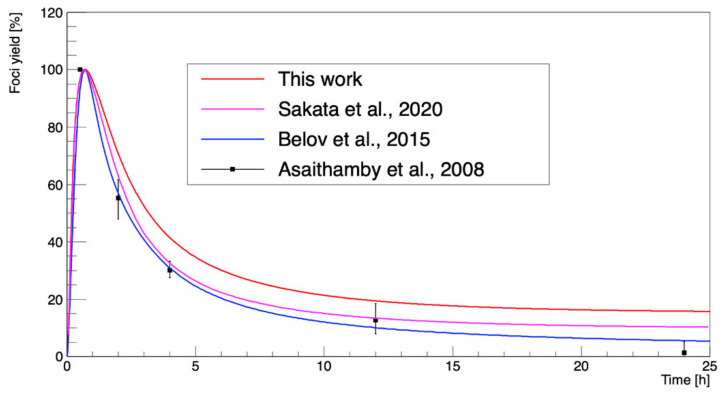
The γ-H2AX yield as a function of repair time from irradiation by ^137^Cs at a dose of 1 Gy. The calculated repair model in this study was compared with the calculations of Belov et al. (2015) [62], Sakata et al. (2020) [15], and the experimental data of Asaithamby et al. (2008) [38].

Figure 7 shows the results of the scaled γ-H2AX yield of ^137^Cs at a dose of 1 Gy as a function of time up to 25 h after irradiation. The results of this work were obtained with the calculated number of DSBs (4.04 Gbp^−^^1^) and the irreparable fraction (~0.15) using the approach described by Belov et al. [62]. The foci yields calculated in this study were larger than in the calculations by Belov et al. [62], Sakata et al. [15], and experimental data [38].

## 4. Discussion

Regarding the results for total strand breaks according to the damage source (Figure 3), the amount of indirect damage decreased as a function of LET, unlike direct damage, because the concentrated molecular species at high LET rapidly recombined with each other, as has been previously reported [58]. Compared to other studies, our results showed less indirect damage yields; on the other hand, the direct damage yields did not change.

These results could be explained by a combination of several factors, such as the initial distribution of radicals determined by our new elastic scattering model, and the revised pre-chemical and chemical parameters, such as the dissociation scheme, including displacement and branching ratio, diffusion coefficients, and reaction rates. In particular, we could explain the difference with Geant4-DNA 2020 [15] because the G-values of ^•^OH radicals (mainly responsible for indirect damage induction) with the new pre-chemical and chemical models were smaller than those seen in Geant4-DNA 2020 [15] and 2017 [16].

All the studies showed direct SB yields induced by physical interactions independent of LET, except Geant4-DNA 2017 [16]. All the Geant4-DNA simulations were smaller than the PARTRAC results because of the smaller effective target volume (*R*_direct_ in this study). Indeed, the new elastic scattering model does not affect direct SB yields.

Quantitative comparison with experimental data remains a challenge because the uncertainties in measurements are still large, owing to the cell cycle, measurement experimental conditions, beam properties, etc. In addition, the experimental data for the SSB and DSB yields were measured with significant variations in the experimental methodology and cell types, as listed in Table 2. For example, in Figure 4, the data from Leloup et al. [44] show relatively increased SSB and DSB yields compared to the other data, apparently due to the influence of base pair density and the histone scavenging effect [15]. Note that the base pair densities of the plasmid, human fibroblast cell, and hamster cell (V79) were 9.4 × 10^−^^6^, 0.012, and 0.015 bp/nm^3^, respectively [36,37,43].

Nevertheless, the main improvement in this work was the better agreement of DSB yields with experimental data for human fibroblast cells (H_Skin_) compared to the other MCTS tools such as Geant4-DNA 2017, 2020, PARTRAC, or KURBUC. This was because the total number of indirect SBs was reduced, owing to the chemistry model used in this study. In addition, the results for alpha particles were in close agreement with the measurements of Hoglund et al. [41].

As underlined in several studies [13,15,22,67], one should keep in mind that the numbers of SSBs and DSBs were very sensitive to the damage scoring parameters, such as the parameters given in Table 1. New and more systematic experimental data on cellular irradiation are clearly needed to better validate the simulations.

For the results on fragment length distribution (Figure 5), we observed that the results in this study overestimated long fragments above 50 kbp as compared to PARTRAC and experimental results, even though the experimental range of the fragment counting method was 23 kbp to 5.7 Mbp [45]. It could be intuitively assumed that a higher number of DSB damages increased the number of fragments at a short fragment length and decreased at a long fragment length if the geometry was the same. For example, the DSB yields of Campa et al. [45] were much lower than those reported by Belli et al. [43], as shown in the top right panel of Figure 4. On the other hand, the PFGE approach subtracted the fragment background; that is, the DNA fragment distribution of unirradiated cells [68]. However, this approach could lead to another systematic uncertainty due to the oversimplification of background fragment distribution [69]. In addition, inter-particle interference could be a reason for this disagreement. In this work, each particle was independent because we assumed radiation-induced DNA damage in the low-dose regions. However, the experimental data of Belli et al. [43] and Campa et al. [45] were for relatively high dose rates of 20 and 3.5 Gy/min, respectively.

Our scavengeable damage results were between those reported by Ito et al. [57] and the data produced by Hirayama et al. [58], as shown in Figure 6. There was no clear reason for this inconsistency, but one hypothesis to explain the observed difference could be that the experimental data of Hirayama et al. [58] from V79 hamster cells were also significantly different from the data generated by Ito et al. [57] as measured in HL-60 human leukemia cells. We speculated that the cell size or base pair density could impact the fraction of indirect damage, as recently reported [70].

In Figure 7, it can be observed that our γ-H2AX yield as a function of time calculated with the model of Belov et al. [62] decayed more slowly than in the results of Asaithamby et al. [38]. We supposed that because the initial DSB yields in this study were relatively lower than the previous ones obtained with Geant4-DNA, the normalized foci yields after the peak were apparently higher. In addition, the work of Belov et al. [62] estimated the irreparable fraction as 0.01, consistent with Asaithamby et al. [38]. Nevertheless, we could simulate the biological stage based on this repair model with strong agreement with the experimental results, within a 10% difference. However, there were still limitations to such an analytical approach; for example, the model was optimized only for human fibroblasts. In addition, this model requires improvement for absolute predictions, because it requires the total number of DSBs and the irreparable fraction as inputs.

## 5. Conclusions

In this study, we verified the plausibility of the “molecularDNA” Geant4-DNA example, which used the IRT approach for the simulation of radiolysis, and overcame the subsequent computational burden. In addition, we applied the developments proposed in our previous works, such as the electron elastic scattering model and pre-chemical and chemical parameters, for the simulation of early DNA damage in a model of a simplified human fibroblast cell nucleus.

We have shown that the results obtained using these improvements were in reasonable agreement with recent experimental data on DSB yields as a function of LET, with acceptable simulation times. To further evaluate the plausibility of our simulations, we also presented various quantities such as SSB/DSB ratio as a function of LET, the distribution of fragment lengths, the scavengeable fraction as a function of LET, and repair of foci as a function of time. These findings underlined the need for more accurate experimental data on DNA damage in irradiated cells.

There were some technical limitations, such as large memory consumption (up to ~160 GBs for only 100 high-LET particles). To overcome such issues, we split the simulation runs.

In the future, further technical optimization of the software will be undertaken to reduce memory consumption. In addition, damage simulations with a more accurate inelastic model of G4EmDNAPhysics_option4 [71,72], instead of the default physics constructor (G4EmDNAPhysics_option2), would generate additional improvements.

The work presented in this study is a step forward in the mechanistic understanding of radiation-induced DNA damage in the cell nucleus. There is still a long way to go before a mechanistic approach is available for use in radiation therapy, which in particular requires prediction of the response of a wide variety of cell lines in tissues. However, the open-code approach described in this article may further help to address this challenge.

## Figures and Tables

**Table 1:** The damage parameters used for predicting direct and indirect DNA damage induction.

Damage Parameters	Values [15]
*R*_direct_	3.5 Å
*E*_lower_	5 eV
*E*_higher_	37.5 eV
*P*_OH_	40.5%
*d*_kill_	9 nm
*T*_chem_	5 ns
*dt*_max_	0.5 ns

**Table 2:** The experimental data and corresponding references, cell lines, cell types, and approaches used for measuring damage.

Reference	Cell Line	Description	Damage Measurement
Frankenberg et al. (1999) [40]	-	Human fibroblasts	PFGE
Hoglund et al. (2000) [41]	GM5758	Human fibroblasts	PFGE
Belli et al. (2000) [42]	V79	Chinese hamster cells	CFGE
Belli et al. (2001) [43]			PFGE
Leloup et al. (2005) [44]	XL2-Blue MRF	Plasmid DNA of bacteria	PFGE
Campa et al. (2005) [45]	AG1522	Human fibroblasts	PFGE
Petkovic et al. (2019) [52]	HTB177	Non-small lung cancer cells	γ-H2AX
Ristic-Fira et al. (2020) [53] Keta et al. (2021) [2]			

**Table 3:** The energies of ^137^Cs, ^60^Co, protons, alphas, and the corresponding LET in water, number of particles, number of runs, and simulation time.

Energy (MeV)	ICRU-90 LET in Water [39] (keV/μm)	Total Number of Particles	Run Splitting	Simulation Time per Run (CPU Hours)
Gamma Rays				
^137^Cs	0.8 ^(a)^	25,000,000 ^(b)^	25	47.43
^60^Co	0.4 ^(a)^	5,000,000	10	56.69
Protons				
0.30	54.41	3000	30	146.90
0.40	46.48	3000	30	101.76
0.70	33.14	3000	30	44.60
1.00	25.77	3600 ^(c)^	36	27.78
1.67	18.12	3000	30	15.46
2.34	14.31	8000	20	100.63
4.00	9.33	10,000	1	435.54
7.00	6.11	10,000	1	654.87
50.00	1.24	10,000	1	25.67
Alphas				
5.00	87.54	100	10	29.67
10.00	52.94	100	10	21.51
15.00	38.96	100	10	12.86

^(a)^ From ICRP-92 report [65]. ^(b)^ Corresponding to approximately 1 Gy dose for reproducing the experimental data of the repair model. ^(c)^ Corresponding to approximately 100 Gy dose for reproducing the experimental data of the fragment distribution.

## Data Availability

The “molecularDNA” application will be distributed through the Geant4 collaboration group as a Geant4-DNA example.
